# Beyond the skin: Stories of advocacy and resilience from women with Hidradenitis Suppurativa

**DOI:** 10.1177/13591053251384387

**Published:** 2025-10-21

**Authors:** Kenzie Tapp, Jasmine Kobrosli, Jessica Kichler, Natalie Giannotti, Kendall Soucie

**Affiliations:** 1 University of Windsor, ON, Canada

**Keywords:** Hidradenitis Suppurativa, resilience, meaning-making, reflexive thematic analysis, feminist social constructionism

## Abstract

Hidradenitis Suppurativa (HS) is a chronic, recurrent inflammatory skin condition that significantly impacts quality of life. The purpose of this qualitative study was to gain a deeper understanding of the lived experiences of women diagnosed with HS by amplifying a more integrative, strength-based understanding of the condition. We explored the lived experiences of 25 Canadian women with HS, focusing on their challenges with HS and how they overcame them through strategies of resilience and meaning-making. Reflexive thematic analysis techniques, situated with a feminist social constructionist lens, generated four major themes: (1) Developing Agency in Symptom Management, (2) Cultivating Methods to Cope, (3) Harnessing One’s Voice for Advocacy and Change, and (4) Gaining Insight and Growth Through Life-Long Learning. This study provides critical insights into improving care and support for women with HS, emphasizing the importance of patient-centered and holistic approaches to HS care that prioritize resilience and address systemic inequities.

Hidradenitis Suppurativa (HS) is a chronic, recurrent inflammatory skin condition categorized by inflamed follicular areas of the body, such as the armpits, groin, and buttocks (Jemec, 2012). HS often manifests as painful nodules, abscesses, sinus tracts, and/or fistulas that follow a cyclical pattern of remission and emergence (i.e. flare-ups; Sabat et al., 2020). Treatment approaches include oral antibiotics and steroid injections, as well as biologics and/or surgical interventions (Altenburg et al., 2011). Globally, HS affects an estimated 1%–4% of the population, with a female-to-male ratio of 3.3:1 (Margesson and Danby, 2014). Common physical comorbidities include obesity, metabolic syndrome, diabetes, arthritis, Crohn’s disease, and polycystic ovary syndrome (Revankar et al., 2021). Individuals with HS may also experience elevated levels of anxiety, depression, and suicidality, as well as body image disturbances and diminished self-esteem (Chernyshov et al., 2021).

Due to the increased incidence rate of HS in women, coupled with the societal and medical expectations surrounding women’s bodies, nearly all comorbidities are heightened in women with HS (Sabat et al., 2020). HS for women is implicitly framed as a condition that disrupts femininity and gender adherence (Fisher and Ziv, 2022). It affects the various social identities and gender roles associated with womanhood, such as being a mother, wife, and provider (Fisher and Ziv, 2022; Revankar et al., 2021). Women tend to experience heightened threats to their gender expression, especially in the context of chronic illness, as their bodies have historically been characterized as their sole way of operating in the world (Hesse-Biber, 2007). This has been further embedded within medical institutions that govern Western societies, which frequently results in women’s symptoms being dismissed, invalidated, and pathologized (Hesse-Biber, 2007; Werner and Malterud, 2003). Thus, this overarching lack of support and judgment can exacerbate the stigma and shame surrounding HS, as well as lead to a distrust of medical institutions and a potential worsening of symptoms, which can negatively impact physical and psychological health outcomes (Krajewski et al., 2021). Much of the current literature surrounding HS, especially regarding women with HS, focuses on these negative aspects, where HS is painted as inevitable and irreconcilable (Chernyshov et al., 2021).

Opposingly, to move away from a deficit-based perspective, there has been a growing interest in examining resilience, especially in relation to the maintenance of chronic conditions (Cal et al., 2015). Resilience encompasses the incidence of bouncing back (Babić et al., 2020), or the ability to foresee challenges and restructure oneself during difficult situations in ways that support and maintain effective functioning (Ungar, 2018). This can be demonstrated in numerous ways, including, but not limited to, active problem-solving, social support, positive coping and/or reframing techniques, religion/spirituality, and seeking professional psychological assistance (Finstad et al., 2021). For individuals with chronic conditions, resilience has been associated with increased treatment adherence, self-empowerment, and overall health-related quality of life, resulting in vast improvements in condition severity, pain, and management (Böell et al., 2016). By fostering a sense of control and capability (Robottom et al., 2012), resilience then offers patients the further possibility of resolution and renewal in the form of growth and meaning-making processes (Park, 2010).

The process of meaning-making depicts how individuals interpret, understand, and make sense of various life occurrences and their concept of self (Park, 2010). This process can lead to personal growth and positive changes in areas such as social relationships, spirituality, coping strategies, resourcefulness, and adherence to treatment (Ferreira-Valente et al., 2021; Park and George, 2013). It is a central aspect in recovering from highly stressful and/or traumatic experiences, such as chronic conditions (Park and George, 2013). Thus, meaning-making further compliments resilience by fostering more transformative change and growth (Elam and Taku, 2022). Together, resilience and meaning-making provide an integrative framework for understanding how individuals navigate the complex challenges of living with HS and how they can engage in adversarial growth.

## Purpose of the study

Currently, HS is framed as having largely negative symptom impacts, which are especially prevalent for women with HS, due to associated violations to femininity in both appearance and social roles. Despite this, women with HS can and do live fulfilling lives with this condition while finding ways to cope with its impacts and navigate oppressive institutions. However, a more strength-based outlook on HS, such as through resilience and meaning-making processes, have yet to be explored within the current literature.

Thus, our aim was to explore the lived experiences of women with HS with a focus on resilience and growth over their lifetimes. Specifically, we sought to understand how women with HS navigate challenges across life domains, identify the strategies they use to manage their symptoms, and explore the broader impacts of HS on their identities and lives as women. We aim to advance this literature by applying a feminist lens to highlight systemic inequities faced by women with HS and using qualitative methods to explore the condition’s psychosocial and experiential impacts. We proposed the following research questions: (a) how do women with HS manage their symptoms and the impacts of the syndrome on their lives, (b) what are the main challenges they face across life domains and how do they navigate them and, (c) what insights have they learned about themselves through these experiences? As a whole, insights from these points of inquiry may help to uncover the strategies that sustain or enhance quality of life in this patient population, which can help to inform further evidence-based strategies, patient care initiatives, and holistic treatment plans to improve the long-term outcomes for women with HS.

## Methodology

### Theoretical framework

According to feminist social constructionism, the main theoretical framework guiding this study, “reality,” and therefore knowledge, is socially constructed (Campbell and Wasco, 2000). Various social factors influence how we view and interpret our realities (Crawford and Marecek, 1989). There is no single reality or truth; rather, multiple truths are constructed through individual and collective experiences (Campbell and Wasco, 2000). Central to this framework is the critique of how these constructs function as mechanisms of oppression (Friedman, 2006). By challenging the notion that such constructs are inevitable and unchanging, this framework seeks to dismantle power imbalances and highlight marginalized voices (Friedman, 2006).

This theoretical positioning intentionally amplifies the voices and experiences of women with HS, whose experiences have been historically relegated to the periphery (Friedman, 2006). It centers women’s voices and perspectives regarding living with HS into the forefront, while also capturing the power struggles and stigma women face from various social constructions. By including strength-based approaches that capture resilience and meaning-making, this framework incorporates alternative perspectives in the understanding and management of this lifelong condition, as well as underscores its implications for health equity.

### Participants

Following clearance from the University of Windsor’s Research Ethics Board (REB#23-085), a total of 25 women diagnosed with HS by a Canadian practitioner were recruited to participate in this study. To be eligible, participants had to be a Canadian or resident of Canada, English-speaking, over the age of 18, self-identify as a woman, and self-report as having received a formal diagnosis of HS from a Canadian practitioner to control for healthcare system differences. Purposeful and maximum variation sampling strategies were employed to ensure that our sample included individuals from diverse social locations and various diagnosis profiles (Ellard-Gray et al., 2015). These strategies included inclusive study advertisements, iterative sampling procedures (e.g. if a particular group was overrepresented in the sample, we used a wait list), and prioritizing interviews with individuals with more diverse demographic identities.

Participants ranged in age from 20 to 63 years old (*M* = 36.16, *SD* = 10.61), with an average time to diagnosis of 11.10 years from the onset of their first symptom(s). This sample consisted of 68% of White women, followed by Black (8%), East Asian (8%), Indigenous (8%), Middle Eastern (8%), South Asian (8%), and Biracial (4%) women. Most participants had completed an undergraduate degree (36%), followed by the completion of a graduate degree (20%), a vocational degree/certificate (20%), a high school diploma or equivalent (12%), some college or university (8%), or some high school (4%). As well, most participants reported part-time employment (i.e. 1–39 hours per week; 52%), followed by full-time employment (i.e. 40 or more hours per week; 32%), self-employment (8%), unemployment and looking for work (4%), and unemployment and not looking for work (4%). Participants were encouraged to select all applicable options when reporting their demographic information to better capture their social identities. Demographic profiles can be found in Table 1.

**Table 1.** Participant demographic profiles.

ID #	Age	Racial/ethnic identity	Severity of HS
01	30	White	Stage 2
02	44	White	Remission
03	43	White	Stage 2
04	30	White	Stage 1
05	30	Middle Eastern & White	Stage 1
06	46	White	Stage 2
07	31	South Asian	Stage 2
08	21	White	Remission
09	20	Black & Middle Eastern	Stage 3
10	50	White	Stage 2
11	54	White	Stage 1
12	63	Indigenous	Stage 2
13	33	White	Remission
14	35	White	Stage 2
15	42	White	Stage 2
16	33	Black	Stage 3
17	30	White	Stage 2
18	43	White	Stage 2
19	43	White	Remission
20	25	White	Stage 3
21	27	South Asian	Stage 1
22	43	East Asian	Stage 1
23	34	Biracial	Stage 2
24	24	Indigenous & White	Stage 2
25	30	East Asian	Stage 1

*Note*. Age is shown in years, and Severity of HS represents the severity at the time of the interview.

### Procedure

To recruit participants, a general study advertisement was circulated through online HS support groups and posted in local dermatology clinics. Interested participants could scan a QR code that linked to a pre-screener survey. Those who completed it were contacted via email by the first author to confirm their interest in participating and to schedule an interview session.

Upon recruitment, participants were provided with an informed consent form and post-study resources. Each participant completed a single, virtual interview session consisting of three components. The interviews were approximately 90 minutes in length and were completed by the first author as no conflicts of interest arose that would have required an alternate interviewer. At the start of the interview, the consent form was reviewed and verbal consent to participate was documented. Second, a semi-structured interview was used to guide the session, which focused on four domains: (1) healthcare experiences; (2) day-to-day experiences managing HS; (3) social support and challenging stigma; and (4) ending reflections, which included holistic understandings of growth and meaning-making (see Supplemental Materials for the interview guide). Lastly, participants were directed to complete a brief online demographic survey to contextualize the sample. Upon completion of the study, participants received a $20 e-gift card to a place of their choice for compensation. All the interviews were orthographically transcribed and assigned a unique identifier code, where any potentially identifying information was removed.

### Approach to data analysis

Braun and Clarke’s (2019, 2021, 2022) reflexive thematic analysis (RTA), situated with a feminist social constructionist lens, was our primary analytical approach. RTA is designed to identify, organize, and interpret patterns of shared meaning across a dataset, with an emphasis on the researcher’s active role in theme development (Braun and Clarke, 2019). Rather than viewing themes as passively “emerging” from the data, RTA treats them as the product of a deeply reflexive, interpretive process, grounded in theory and context (Braun and Clarke, 2019). This process is recursive and non-linear, allowing for flexibility in exploring complex themes while remaining grounded in the data (Braun and Clarke, 2019, 2022). The analytical team included the principal investigator (KT), a research assistant (JK), and a research supervisor (KS).

The analysis followed six phases. First, KT, and JK reviewed all the interview transcripts, while listening to their corresponding audio files, and documented initial reflections in separate research journals. In phase two, they completed line-by-line coding to extract units of meaning that were relevant to the research questions. These codes were not fixed or objectively extracted but reflected initial interpretations shaped by the researchers’ reflexive engagement. In phase three, KT and JK examined codes for shared meanings and began developing preliminary themes. This process was iterative, as considerations were made about how accurately the themes represented an organizing concept, related to the research questions, and connected to the other themes. In phase four, KT, JK, and KS reviewed the themes against the transcripts to confirm their validity and coherence, ensuring they captured participants’ stories. KT, JK, and KS then collaboratively defined and named the themes, summarizing their core concepts with written descriptions in phase five. In phase six, themes were structured into a cohesive narrative with supporting quotes, which was discussed among all authors (KT, JK, JCK, NG, KS) to reach consensus. All authors engaged in collaborative discussions throughout to refine themes and ensure consistent interpretation.

### Trustworthiness

To establish credibility, dependability, confirmability, transferability, and authenticity, rigorous qualitative research standards by Lincoln and Guba (1985) were implemented. This included the analytical team documenting their reflexive notes in research journals; conducting debriefing sessions to review progress and discuss feedback; maintaining audit trails and peer debriefing logs to document discussions; involving multiple coders to safeguard agreement; selecting exemplary quotes to best represent the themes to illustrate an overall narrative; and transparently reporting on the context, location, participants, and methods of analyses. This study also adhered to principles of reflexivity, acknowledging that the findings represent a co-construction of meaning between the participants and the research team (Dodgson, 2019). Each author’s positionality was considered throughout the analysis to mitigate bias and deepen analytical insights (see Supplemental Files for positionality statements).

## Results

### Developing agency in symptom management

This theme highlights participants’ struggles to receive adequate care from healthcare practitioners, often leading them to self-manage their symptoms. Many described feeling unsupported, lost, and confused, as they were frequently left without any guidance or support from practitioners. Over time, this fostered a sense of empowerment and greater bodily awareness.

#### Becoming in-tune with the body

Following diagnosis, some participants expressed a renewed trust in their bodies, recognizing they had sensed something was wrong all along. For example, P01 stated, “I learned that I know my body best. And all those doctors or nurses saying, ‘It’s nothing. It’s hormones.’ The fact that I kept it in the back of my mind, ‘No, this is worse than everyone’s saying it is.’” P05 reflected this sentiment as well:

So, I think one of the biggest things is that despite feeling like they kind of broke my trust in a way, had it not been for those experiences, I wouldn’t have found myself in a place where I actually trust myself more and listen to my body.

This closer bond permitted these participants to recognize how their bodies were doing their best in managing their symptoms, challenging the belief that their bodies were failing or working against them. They reflected on how they appreciated these signals. For instance, P05 articulated her deep appreciation for her body’s signals:

And another thing I’ve learned from the experience is I’ve opened up dialogue with my body . . . Because once you start shutting that communication down, things become a little more unpredictable . . . So, it’s like my body has a bigger voice now.

Participants felt reassured knowing that their bodies were “looking out” (P05) for them, where they felt a greater sense of confidence in their body’s ability to manage the uncertainty of HS. However, they also recognized a balance with this mindset, where they acknowledged that sometimes their bodies “can only do so much” (P11) and accepted this messaging to find a “better balance” (P05) in their lives.

#### Regaining a sense of “control.”

Some participants explained how they “took back the reins” (P06) by taking ownership of their care and initiating their own research on symptom management. For example, P21 explained that “a lot of the relief that [she has] now is because of things that [she] had to figure out on [her] own.” P13 also added: “For me, I’ve just really learned to take your health into your own hands . . . and also, just do your own research. Because there’s so much on the Internet that you can find on it or realize.”

They also emphasized testing the effectiveness of these techniques. Once they found the methods that bettered their HS, they constructed a management hierarchy where they would approach their symptom presentations in a stepwise manner. This empowered participants to feel more at ease when experiencing flare-ups, as they felt reassured by having a plan in place. This was reflected by P24 where she described having a “routine” to tackle her symptom presentations before letting herself “spiral” and P02 who said she “finds refuge in making it a routine.”

Despite re-establishing a sense of control, they also maintained a realistic view of what this “control” truly meant when living with an unpredictable condition like HS. They acknowledged that they did not need to, nor could they have, complete control over their HS, and were comfortable with it’s “ebbs and flows” (P05). Ultimately, participants redefined “control” in relation to HS, gaining a sense of authority over their condition.

### Cultivating methods to cope

To better manage their HS symptoms beyond Western medicine and in response to limited healthcare support, some participants adopted a range of coping strategies. They formed a toolbox of strategies, which prevented them from engaging in negative, self-perpetuating cycles of worry, frustration, and hopelessness. Their approach was often holistic, reflecting elements of a biopsychosocial model of support, even if applied unconsciously.

#### Engaging social supports

Most participants described how important it was to develop and rely on close social supports during more difficult times with their HS. They explained how conversations with others provided them with a sense of “validation” (P01) and “relief” (P01). P23 further explained how important her family’s support was during difficult times with her HS:

I guess what brings me a little bit of peace is knowing that my family is here if I really need to take care of me . . . Knowing that although it’s me, that I have all these symptoms, I feel deep inside of me that I’m not alone.

They also frequently found care and support through connecting with others with HS, as well as those with other chronic health conditions, often through online communities. They described how important it was to connect with others who are “going through the same thing as [them]” (P07) to feel “not so alone” (P07) in managing their condition. These connections led these participants to feel more hopeful about their future with HS.

#### Engaging the body

Some participants described how they used physical activity to relieve their symptoms, though accessibility varied. For example, P07 stated, “If you’re feeling miserable all the time, it’s just like, go for a walk.” P24 also added how she modifies her exercise needs based on her HS symptoms to ensure she still receives the benefits of physical movement, “I guess adjust based on the symptoms that are present. Whether that be clothes or not going to the gym that day and going swimming instead or doing different things.”

Participants emphasized the importance of “prioritizing [themselves]” (P13) through enjoyable movement. Those who engaged in physical activity noted it “was the best for [them]” (P23), adapting to support this need despite HS’s unpredictability. Though, at times, HS made it difficult for participants to be physically active, they acknowledged how important it was for their physical symptoms and mental well-being.

#### Engaging psychological practices

Many participants adopted cognitive strategies to ease the psychological impacts of HS. During flare-ups, they reminded themselves that their HS symptoms were temporary. They described challenging their negative thoughts by recalling that their condition “won’t last forever” (P11) and they needed to “wait it out” (P03).

Some also reframed their mindset by focusing on positives in their lives and appreciating when they had good days with their HS symptoms. For example, P09 explained, “And so I guess being able to accept the fact that I do have HS but also being able to know that there are good moments and there are good days. And I’ve experienced those fully.” P03 illuminated this further by stating:

If something happens, I’ll be like, “Okay, so this is happening. Let’s think of three ways this could be worse . . . And you know what? Even though it hurts, for the most part, I am healthy. . .” So, I find that kind of grounds me a bit.

In relation to this, some participants also used humor to reframe their HS experiences in a more positive light. They used humor not only to cope personally, but also to “normalize” (P02) their condition and show others that their “life doesn’t end because of this” (P02). P05 stated, “I kind of lean on laughter as a way to kind of reframe an [HS] experience where, had it not been for this, we wouldn’t be laughing.”

To avoid “spiraling” (P01) about their flare-ups, participants emphasized staying present. They used phrases, such as “one step at a time” (P02) or “cross that bridge when you get there” (P03) to stay grounded. At times, they also deliberately ignored their HS-related worries to better attend to immediate tasks.

Participants also turned to formal mental health strategies. They cited techniques such as deep breathing, the 5-4-3-2-1 grounding method, guided meditation, and gratitude journaling. Additionally, some sought therapy services to better process and understand their experiences, something they found difficult to do alone.

Overall, participants showed resourcefulness in identifying different psychological strategies that helped them to cope with their condition. They used a trial-and-error process to discover what worked best, recognizing the importance of caring for themselves across various dimensions. Many found that their HS became more manageable when they approached their needs holistically.

### Harnessing one’s voice for advocacy and change

This theme highlights how some participants sought strategies to advocate for themselves and their HS to receive better support and incite change. This was often in response to an initial lack of appropriate solutions from the healthcare system and the challenges they faced navigating it. Many realized that they needed to be their strongest advocates, as only they could truly identify their needs and potentially advance HS care and research.

#### Asserting one’s needs

Some participants realized that to receive better care from medical professionals and close others, they needed to voice their needs and push back against resistance. In healthcare settings, participants prepared for their appointments by making notes, outlining talking points, and using their own research to inform their medical decisions. When faced with limited support from providers, they described pushing back and not leaving until they got an answer. As P08 explained, “There is a time when you gotta fight for yourself. I feel that no one is going to look out for my health but me,” while P21 shared the following sentiment, “Vouch for yourself. You’re own spokesperson. You’re your own hero. You’re your own everything.”

This advocacy extended beyond healthcare spaces and into personal relationships. Participants described informing their social circles about their HS to gain support. When met with confusion of dismissal, they reiterated their needs and set boundaries to protect their well-being. For example, P07 described how she communicates her boundaries to her friends during a flare-up: “If I can’t do it, I just say, ‘Please don’t take it personal. This is what I kind of have to follow for my life to be normal and I need you to respect that.’” Across contexts, participants emphasized reclaiming their voice as key to having their HS needs recognized and respected.

#### Challenging stigma and societal norms

Additionally, HS prompted many participants to confront societal “rules” (P25) surrounding womanhood and how these norms shaped their symptom management. This was often tied to stigma, especially in relation to weight, hair growth, and scarring. In response, participants challenged the implicit expectations placed on them rather than passively accepting them. For example, P11 resisted weight-related stigma from her family members who assumed that weight loss would improve her HS:

I think there’s obviously lots of stigma and lots of embarrassment, and it’s related to weight . . . You know, some of the stuff talks about, “If you just lose weight, it gets better.” And it’s like, “Well, I was a lot thinner, and it wasn’t better.” So, no thanks.

P03 similarly questioned the labels often assigned to specific body types, especially those stigmatized in HS discourse:

I am a person who is fat. And oh, three years ago, I would have never said I’m a person who’s fat . . . Now I’m like, “No. Short, fat, tall, skinny, bald. It’s just a label. Whatever.” So, I’ve really done a lot of work on trying to let go of what other people may think of me. I can’t control what people think of me, so why waste energy on that?

To further communicate and stand up for their symptom management decisions, especially those that were more cosmetic in nature, some participants began to openly challenge gender norms. This was expressed by wearing certain clothing to “show off” their “hairy armpits” or express how “sexy” they felt in loose clothes that did not exacerbate their HS, finding a balance between self-expression, confidence, and comfort (P04). P04 reflected:

But that really has helped me just realize too that everything is all about society . . . So now, I’m kind of pushing the boundaries to show everyone that this is how society has made it seem. And as well too . . . we might think, “women should do this.” And that whole “should” thing. Where did we get that “should” thing from? Like, there’s no such thing as a “should,” right?

Others reflected on the deeper meaning behind societal messages and worked to reframe the idea of being seen as “abnormal.” This led to a re-evaluation of the societal value placed on appearance and a subsequent re-shaping of personal values. For instance, P05 realized that she has “much more to offer than [her] appearance.” P16 further echoed this sentiment:

I think it’s made me not as superficial. Nothing about looks and all of that. It’s just that those really aren’t my priorities anymore. I’d rather just be a caring and compassionate person . . . instead of always worrying about how you look or how others look and judging them off that.

#### Passing on knowledge to others

Participants used their voices and newfound insight to speak more openly about their experiences, aiming to educate others and spread awareness about HS. This typically occurred in personal settings where they knew people better, such as at work, school, and family/peer events. However, a few participants felt quite open and shared information about HS in any setting they felt they needed/wanted to. For instance, P20 explained how she carries around a book about HS with her and uses it to broach conversations with people about HS because “if they don’t understand” she’s “gonna educate them about it.”

Many participants chose to be more open to support others with HS, especially future generations, in hopes of preventing similar struggles. Helping others not only assisted others with their HS, but it also gave participants a sense of purpose. P03 described how impactful this was for her:

I’m not afraid to talk about my HS. I’m an open book. So, I post on Facebook during Awareness Week, even when it’s not Awareness Week . . . And actually because of that, I’m very proud to say that a friend of mine, her daughter was showing symptoms, and sure enough, she has HS . . . So, I’m very glad that I’m able to help raise awareness about HS and hopefully help people understand what’s going on with their bodies. Perhaps a little sooner than they might have otherwise.

Being vocal about HS helped participants feel purposeful and fostered a sense of advocacy, while also supporting others in similar situations. They noticed the “ripple effect,” (P13) which motivated them to continue to share their HS stories. They believed their openness could help advance HS research and care, and potentially reduce misdiagnoses and underdiagnoses.

### Gaining Insight and growth through life-long learning

As participants became more equipped to manage, cope with, and advocate for their HS symptoms, some described finding meaning and personal lessons within their diagnostic experiences. Despite ongoing challenges with their condition, they often expressed gratitude for the lessons HS taught them.

#### Journey to self-acceptance

Some participants accepted their bodies for how they were, including their HS symptoms, and began to engage in self-love and appreciation. This shift began by releasing their self-blame and guilt related to their HS. They expressed how receiving a diagnostic label of HS was important to begin this process, and how this provided a sense of relief that there was a medical reason behind their symptoms. For example, P01 stated, “I think just talking about it or putting a name to it and saying, ‘This is a flare up’ or ‘I am in pain’ or kind of separating it from me and saying, ‘It’s not my fault.’ Again, taking away the blame, there’s a reason for this.”

With the guilt and self-blame reduced, some participants were then able to show more kindness toward themselves during difficult moments. They described learning to “give [themselves] grace” (P03) when they are having a difficult time with their HS and are generally more understanding toward themselves. They explained that they “try not to force [themselves] through anything” (P08) and give themselves “twice the extra grace to focus on [themselves]” (P07).

This shift supported a greater acceptance of HS and how they could live with it in a harmonious way. Participants described their HS as “a part of [their] life, [their] stories, and who [they are]” (P13). This helped them to cope with the physical changes caused by HS and pushed them to move beyond feelings of discomfort or shame. For example, P20 stated:

I don’t feel like there’s anything I can do to change the situation or what my body looks like. So, I try to be accepting of it as much as I can . . . I just try to tell myself my body is fine the way it is, you know? Can’t really change the scars or the abscesses or anything about it. It just is what it is. . .I just try to be my best self.

This bodily acceptance, once experienced with HS, often pushed them to apply this to other aspects of their appearance that were not necessarily directly caused by HS. They began to be “comfortable with [themselves]” (P04), “gain more confidence back” (P15), and “wholeheartedly love [themselves]” (P25). P04 expressed how impactful her HS was on this journey of self-love and acceptance:

Without HS, like I said, not that I was like, “Oh, yay! Having HS is a good thing,” but without it, I would probably still be like, “Oh, you know, I don’t look sexy and skinny, like everyone else.” But from just having HS, like weight, I was like, “Okay, that’s not even something on my radar anymore” . . . I just embrace everything.

#### Deeper empathy toward others

Some participants not only developed acceptance and kindness toward themselves, but they also applied this to others. They described how their experiences with HS led them to have a greater understanding of chronic health conditions, where they developed more compassion and empathy toward those with such conditions. They stated that they were able to “fully see other people’s experiences in ways that maybe people without chronic conditions might not necessarily see” (P05). More specifically, the unseen nature of HS made them appreciate “hidden disabilities or conditions” more (P15).

Some participants explained how this lesson of greater empathy extended beyond those with chronic illnesses to broader social interactions. For instance, P13 explained how she has used this teaching with her employees, which has strengthened the team dynamic at her workplace, whereas P14 described how she fosters this perspective with her friends and family and is known as the “person to go to” for support. They highlighted that “you never know what someone is going through” (P20) and so it is “always better to approach things with love and kindness” (P14).

#### Newfound strength

Lastly, through the challenges they faced with their HS, participants reflected on their inner strength required to persevere through such adversity, which helped them adopt a more positive outlook on future difficulties. They described this mindset as a “superpower” (P21); they felt that they truly embodied what it meant to be a “HS warrior” (P01). More specifically, P20 stated, “I learned that I must be pretty damn strong if I can make it through those bad days,” while P02 said “[HS] just taught me I’m a pretty strong person, strong personality, and nothing’s gonna take me down.”

This strength reduced anxiety about the future, not only regarding their HS, but also about other challenges that could come their way. They felt like, “HS is the toughest thing [they’ve] had to deal with so far” (P16), where they “can overcome anything that happens . . . if [they] just hang in there” (P13).

With this newfound strength in mind, participants felt more equipped and at-ease when thinking about the uncertainty of their HS, which often resulted in more success with managing their symptoms. They were no longer consumed by the chronic nature of their HS, despite the messaging that was presented to them. Instead, they became more active problem solvers, where they sought solutions rather than succumbing to negative emotions.

## Discussion

This study provides novel insights into how women navigate the challenges of living with HS within oppressive contexts. By highlighting resilience, meaning-making, and growth, our findings offer a more holistic, strength-based understanding of HS. Our insights are derived directly from patient’s experiences, which provides fruitful avenues for change. Four themes characterized participants’ lived experiences, including (1) Developing Agency in Symptom Management, (2) Cultivating Methods to Cope, (3) Harnessing One’s Voice for Advocacy and Change, and (4) Gaining Insight and Growth Through Life-Long Learning.

Participants’ journeys often began with feeling isolated and inadequately supported by the healthcare system. This lack of guidance often pushed many to take initiative in understanding and managing their condition. This manifested through two interconnected sub-themes, including (1) becoming more in-tune with their bodies and (2) finding more balanced control over their HS. The latter sub-theme aligns with existing literature on the benefits of accepting chronic conditions through the concept of flexible control to achieve better health outcomes (Stewart and Yuen, 2011). However, the former finding—that developing trust in one’s body fosters resilience—provides a nuanced perspective. While chronic illness literature often highlights disruptions in bodily trust (Charmaz, 1995), this study underscores the potential for body connectedness to contribute to growth and empowerment (Cooper et al., 2010; Herre et al., 2016). Women in this study reframed their bodies as partners in their symptom management, rejecting narratives that cast their bodies as broken, which represents a novel avenue for fostering resilience in women with HS.

Additionally, the concepts of advocacy and generativity were also noted amongst some participants, and although these concepts are documented within the current literature (e.g. Babić et al., 2020), they remain underexplored in HS-specific research. Gendered dynamics often compounded our participants’ experiences, where women in our sample felt that it was their responsibility to make social contributions to aid future generations so that they would not have to experience the same oppressive systems that halted their care. Incorporating the intersection of gender to explore these concepts is largely lacking in HS research. This lens is crucial as women report needing to increase their advocacy efforts to push back against oppressive systems and institutions within society (Werner and Malterud, 2003).

Participants also actively challenged societal norms regarding their “abnormal” bodies and their subsequent violation of Western feminine ideals. It should not go unnoticed though that the adherence to social and bodily norms and expectations were still very present amongst our participants’ accounts. For example, they were often bombarded by the social norm to be physically active while living with a chronic health condition, the gender norm that assigns women with the charge of educating people in their social circles and taking care of their emotions and feelings, and the expectation that women need to be “warriors” within such complex circumstances. Complying or grappling with such normative standards might lead to an additional burden that could complicate or impede the daily management of HS for women (Fisher and Ziv, 2022). Continuing to ignore this perspective in the literature perpetuates the burden placed on women with HS and limits the development of supportive interventions to better assist them with overcoming these major pervasive stressors.

Lastly, some participants described elements of meaning-making and growth, which were consistent with findings from other chronic illness populations (Ching et al., 2012; Rashidi et al., 2021). However, it should be explicitly noted that resilience and meaning-making are socially defined and distributed constructs, where members of marginalized groups across various intersections may experience more difficulties achieving this outcome due to layered social and systemic barriers (Walker and Peterson, 2018). As such, some women with HS are more likely to experience compounded challenges due to their social positionings and systemic inequities (Adler and Schwaba, 2024). Ultimately, it is important to acknowledge that some women with HS may be better positioned to access resources and engage in opportunities to foster growth, while others may have additional burdens to overcome (Adler and Schwaba, 2024). Our findings reinforce critiques of resilience as an individual trait, emphasizing the need for systemic support to mitigate barriers and foster equitable outcomes (King et al., 2024).

### Implications

The findings from this study provide actionable insights for promoting resilience and improving care for women with HS. By identifying the antecedents of more favorable outcomes, this study offers a more prevention-based approach instead of the correction-based approach that dominates current Western medical practices (Aizpurua-Perez and Perez-Tejada, 2020; Trivedi et al., 2011). The strategies identified by participants offer a foundation for developing resilience-focused interventions, such as tools to promote body connectedness, coping strategies, and advocacy skills. Improving resilience within this HS sub-population, and especially those from diverse social locations, should be an essential consideration in optimizing HS care and patient outcomes.

However, the onus should not be placed solely on the patient to exhibit resilience in the face of a broken social system. Rather, corrective-based strategies to address the issues within these systems should be the primary focus so that there are less inequities or barriers for women with HS to navigate and overcome. More comprehensive guidance and support at the time of diagnosis may be beneficial, which could be achieved through individualized/personalized care plans (Edwards et al., 2017). The coping strategies and resilience practices identified by participants of this study provide an initial conceptualization of the skills, supports, and resources that could inform various patient materials. Additionally, using an interdisciplinary team approach to address patients’ diverse needs can promote psychological well-being alongside physical health management.

Moreover, education about the psychosocial impacts of HS within the medical community could help to better identify and address patient needs. This could include workshops on implicit bias, empathy, communication, and patient-centered care, while also integrating an intersectional lens to capture the nuanced experiences of HS patients. Acknowledging and providing assistance or resources to foster systemic reform should be paramount to prioritize equitable access to care. Strategies could include providing/sourcing interpreters, extending clinic hours, conducting social history assessments, and/or referring patients to specialized support services. By addressing these systemic gaps, the healthcare system can better meet the needs of women with HS and reduce the fragmented care and delays reported by our participants.

### Limitations and future directions

This study’s primary limitation was the lack of demographic diversity, with the sample skewed predominantly toward white, middle-class, and educated individuals. This homogeneity limited the deeper exploration of intersectional perspectives, which are integral to a feminist social constructionist lens (Friedman, 2006). Future research should employ further strategies to recruit more diverse samples to better capture the unique experiences of marginalized groups with HS.

Additionally, recruitment through online HS forums may have introduced bias, potentially attracting a particularly resilient sample, as individuals in these spaces are often actively seeking support. Desirability bias may also have influenced participants to emphasize positive experiences, given the study’s focus on resilience. Future studies could address this by recruiting from broader community-based settings and exploring both resilience and vulnerability. Despite these limitations, the findings highlight the importance of integrating strength-based and systemic approaches into HS care to promote more equitable and effective support for this population.

## Supplemental Material

sj-jpeg-1-hpq-10.1177_13591053251384387 – Supplemental material for Beyond the skin: Stories of advocacy and resilience from women with Hidradenitis Suppurativa Supplemental material, sj-jpeg-1-hpq-10.1177_13591053251384387 for Beyond the skin: Stories of advocacy and resilience from women with Hidradenitis Suppurativa by Kenzie Tapp, Jasmine Kobrosli, Jessica Kichler, Natalie Giannotti and Kendall Soucie in Journal of Health Psychology
