# Supplementary figures and images for: Beyond the skin: Stories of advocacy and resilience from women with Hidradenitis Suppurativa
Source: J Health Psychol. 2025 Oct 21;31(6):2300–13. doi: 10.1177/13591053251384387 (PMC13070130; doi:10.1177/13591053251384387)

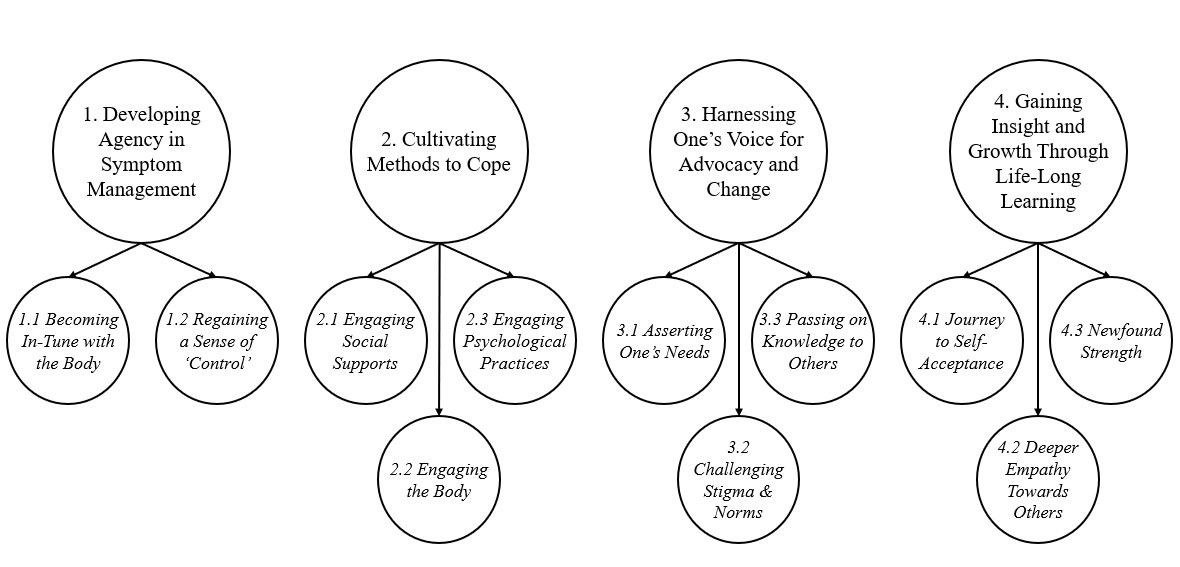

Supplement: sj-jpeg-1-hpq-10.1177_13591053251384387 – Supplemental material for Beyond the skin: Stories of advocacy and resilience from women with Hidradenitis Suppurativa [file sj-jpeg-1-hpq-10.1177_13591053251384387.jpeg]
